# Enhancing the Accuracy of Human Phenotype Ontology Identification: Comparative Evaluation of Multimodal Large Language Models

**DOI:** 10.2196/73233

**Published:** 2025-06-02

**Authors:** Wei Zhong, Mingyue Sun, Shun Yao, YiFan Liu, Dingchuan Peng, Yan Liu, Kai Yang, HuiMin Gao, HuiHui Yan, WenJing Hao, YouSheng Yan, ChengHong Yin

**Affiliations:** 1 Department of Prenatal Diagnosis, Beijing Obstetrics and Gynecology Hospital, Capital Medical University, Beijing Maternal and Child Health Care Hospital, 251 Yaojiayuan Road, Chaoyang District, Beijing, 100020, China, 86 15572779093; 2 Department of Reproductive Medicine, Shijiazhuang People's Hospital, Hebei Province, Shijiazhuang, China; 3 Department of Gynecology and Obstetrics, Yijishan Hospital of Wannan Medical College, Anhui province, Wuhu, China; 4 School of Medicine, South China University of Technology, Guangdong Province, Guangzhou, China

**Keywords:** multimodal large language models, ChatGPT, rare diseases, human phenotype ontology, open-source LLMs, large language model

## Abstract

**Background:**

Identifying Human Phenotype Ontology (HPO) terms is crucial for diagnosing and managing rare diseases. However, clinicians, especially junior physicians, often face challenges due to the complexity of describing patient phenotypes accurately. Traditional manual search methods using HPO databases are time-consuming and prone to errors.

**Objective:**

The aim of the study is to investigate whether the use of multimodal large language models (MLLMs) can improve the accuracy of junior physicians in identifying HPO terms from patient images related to rare diseases.

**Methods:**

In total, 20 junior physicians from 10 specialties participated. Each physician evaluated 27 patient images sourced from publicly available literature, with phenotypes relevant to rare diseases listed in the Chinese Rare Disease Catalogue. The study was divided into 2 groups: the manual search group relied on the Chinese Human Phenotype Ontology website, while the MLLM-assisted group used an electronic questionnaire that included HPO terms preidentified by ChatGPT-4o as prompts, followed by a search using the Chinese Human Phenotype Ontology. The primary outcome was the accuracy of HPO identification, defined as the proportion of correctly identified HPO terms compared to a standard set determined by an expert panel. Additionally, the accuracy of outputs from ChatGPT-4o and 2 open-source MLLMs (Llama3.2:11b and Llama3.2:90b) was evaluated using the same criteria, with hallucinations for each model documented separately. Furthermore, participating physicians completed an additional electronic questionnaire regarding their rare disease background to identify factors affecting their ability to accurately describe patient images using standardized HPO terms.

**Results:**

A total of 270 descriptions were evaluated per group. The MLLM-assisted group achieved a significantly higher accuracy rate of 67.4% (182/270) compared to 20.4% (55/270) in the manual group (relative risk 3.31, 95% CI 2.58‐4.25; *P*<.001). The MLLM-assisted group demonstrated consistent performance across departments, whereas the manual group exhibited greater variability. Among standalone MLLMs, ChatGPT-4o achieved an accuracy of 48% (13/27), while the open-source models Llama3.2:11b and Llama3.2:90b achieved 15% (4/27) and 18% (5/27), respectively. However, MLLMs exhibited a high hallucination rate, frequently generating HPO terms with incorrect IDs or entirely fabricated content. Specifically, ChatGPT-4o, Llama3.2:11b, and Llama3.2:90b generated incorrect IDs in 57.3% (67/117), 98% (62/63), and 82% (46/56) of cases, respectively, and fabricated terms in 34.2% (40/117), 41% (26/63), and 32% (18/56) of cases, respectively. Additionally, a survey on the rare disease knowledge of junior physicians suggests that participation in rare disease and genetic disease training may enhance the performance of some physicians.

**Conclusions:**

The integration of MLLMs into clinical workflows significantly enhances the accuracy of HPO identification by junior physicians, offering promising potential to improve the diagnosis of rare diseases and standardize phenotype descriptions in medical research. However, the notable hallucination rate observed in MLLMs underscores the necessity for further refinement and rigorous validation before widespread adoption in clinical practice.

## Introduction

The Human Phenotype Ontology (HPO) is a comprehensive and standardized vocabulary designed to describe phenotypic abnormalities associated with over 8100 diseases [1]. It has become the de facto standard for deep phenotyping in rare diseases and is widely used by researchers, clinicians, informaticians, and electronic health record systems globally [1-4]. The HPO’s detailed descriptions and computable disease definitions enhance diagnostic accuracy, especially when integrated with model organism data [5-7]. It is also a core component of tools like Face2Gene [8] and Exomiser [9], which identify disease-causing variants from sequencing data [1]. The HPO’s interoperability enables integration with other ontologies, advancing genomics and phenomics research [1,10]. As the HPO evolves, its user base grows, and the project team continually expands its content, language translations, mappings, and computational tools to meet increasing demands [6].

Despite the significant utility of the HPO in clinical and research settings, its practical application faces several challenges. First, the HPO includes over 18,000 terms and more than 156,000 annotations for genetic disorders [1], organized in a logically structured hierarchy with the most specific terms at the periphery. This complex framework makes it difficult for clinicians and researchers to fully understand and accurately apply all terms, increasing the risk of omissions or misapplications. Second, the presence of semantically similar terms and synonyms within the HPO complicates term identification and matching, further hindering its use. Most notably, even with user-friendly web-based interfaces [11], describing patients’ abnormal phenotypes using standardized HPO terms remains a significant challenge for less experienced physicians. This difficulty arises from variations in language use and the inherent complexity of human anatomy [2,6]. These barriers limit the broader adoption of the HPO in electronic health record systems and research publications, potentially restricting its impact on advancing rare disease diagnosis and precision medicine. Inadequate patient phenotyping and inaccuracies in clinical descriptions are key factors contributing to the prolonged diagnostic odyssey faced by many individuals with rare diseases, often requiring years and consultations with multiple specialists to achieve an accurate diagnosis [12-15].

Large language model (LLM), exemplified by ChatGPT, has drawn significant attention since their public release in 2022, heralded as catalysts for the fourth industrial revolution [16-18]. These models can respond to free-text queries without task-specific training, sparking both excitement and concern about their potential use in health care [17,19]. Initially designed for text-based tasks, LLMs have shown promising but inconsistent performance across various medical applications [20-26]. With technological advancements, models like ChatGPT have improved their ability to generate high-quality responses comparable to those of experienced medical professionals [17,27-31]. However, their reliance on text-only processing remains a limitation for addressing comprehensive medical scenarios.

In recent years, multimodal large language models (MLLMs) have emerged as a significant advancement in natural language processing and computer vision, demonstrating substantial potential for integrating medical image and text analysis. For example, studies using ChatGPT-4o, the latest multimodal version of ChatGPT, have shown high diagnostic accuracy with text and image inputs, outperforming medical students on *New England Journal of Medicine* Image Challenge cases [32]. However, evaluations of ChatGPT on the Japanese National Medical Licensing Examination highlight ongoing challenges in achieving adequate diagnostic accuracy [33]. Overall, research on applying MLLMs to medical image analysis remains limited [18,33]. The potential of MLLMs to integrate images and text opens new possibilities for richer medical applications, including the identification of HPO terms from patient images. We propose that investigating MLLMs in HPO description analysis will not only advance automated term recognition and semantic matching in clinical HPO applications but also significantly contribute to the evolution of precision medicine, improving diagnostic accuracy and therapeutic strategies for rare disease management. The innovation of this study lies in advancing the application of LLMs in medicine from pure text to multimodal integration, addressing the critical challenge of accurately describing human phenotypes using HPO terms in clinical practice.

## Methods

### Patient Phenotype Images

The Chinese Rare Disease Catalogue includes 207 rare diseases [34], selected by the Chinese government based on criteria such as incidence rate, disease severity, and diagnostic clarity. Most of these diseases are genetic, making the catalog a valuable resource for studying clinically significant disease phenotypes. Patient images were sourced from the Open-i [35], using disease names from the catalog. Inclusion criteria were (1) high image quality, ensuring clarity; (2) a distinct presentation of phenotypic features; (3) the presence of a caption describing the patient’s phenotype; and (4) relevance of the depicted phenotype to the searched disease. A total of 27 images meeting these criteria were included, each displaying abnormal physical characteristics associated with the respective diseases. Web links to the images are provided in Sheet 1 in Multimedia Appendix 1. Some images were cropped to highlight relevant phenotypic features.

### Ethical Considerations

This study used publicly available patient images from the internet for descriptive analysis of features. No processing was performed on the images, and the manuscript does not contain any identifiable patient images. As a simulation of clinical diagnostic trials without intervention on real patients, this study complies with the institutional guidelines for ethics committee exemption.

### Phenotype Recognition Using MLLMs

To identify phenotypes in the selected patient images, we initially used ChatGPT-4o using the following prompt:

*Now I will provide you with pictures of patients from open access literature, without involving patient privacy. You need to identify the content of the picture and answer which of the most obvious Human Phenotype Ontology (HPO) terms are shown in the patient in this picture. Each picture may contain one or more HPO terms. Only answer the most obvious ones, and do not answer the HPO terms you cannot judge. The answer needs to include the names of the HPO terms, and explain and attach the HPO ID*.

As the research team’s native language is Chinese, all interactions with ChatGPT-4o were conducted in Chinese. For each image, the command “please describe the most prominent HPO terms in this picture” was used. In specific cases, such as an image of a patient with albinism, contextual details (eg, the patient’s Asian descent) were provided to ensure accurate phenotype recognition.

To further evaluate phenotype recognition capabilities, we tested 2 open-source MLLMs developed by Meta—Llama3.2:11b and Llama3.2:90b. These models were deployed locally using the Ollama inference framework (version 0.5.10; Ollama) and Cherry Studio (version 1.0.0; Shanghai Qianhui Technology), a user-friendly software for model operation. The recognition process for them followed the same protocol as ChatGPT-4o. Complete chat logs, including prompts, responses, and identified HPO terms, are available in Sheet 2 in Multimedia Appendix 1.

### Study Design

#### Junior Physicians Recruitment

**Figure 1.**
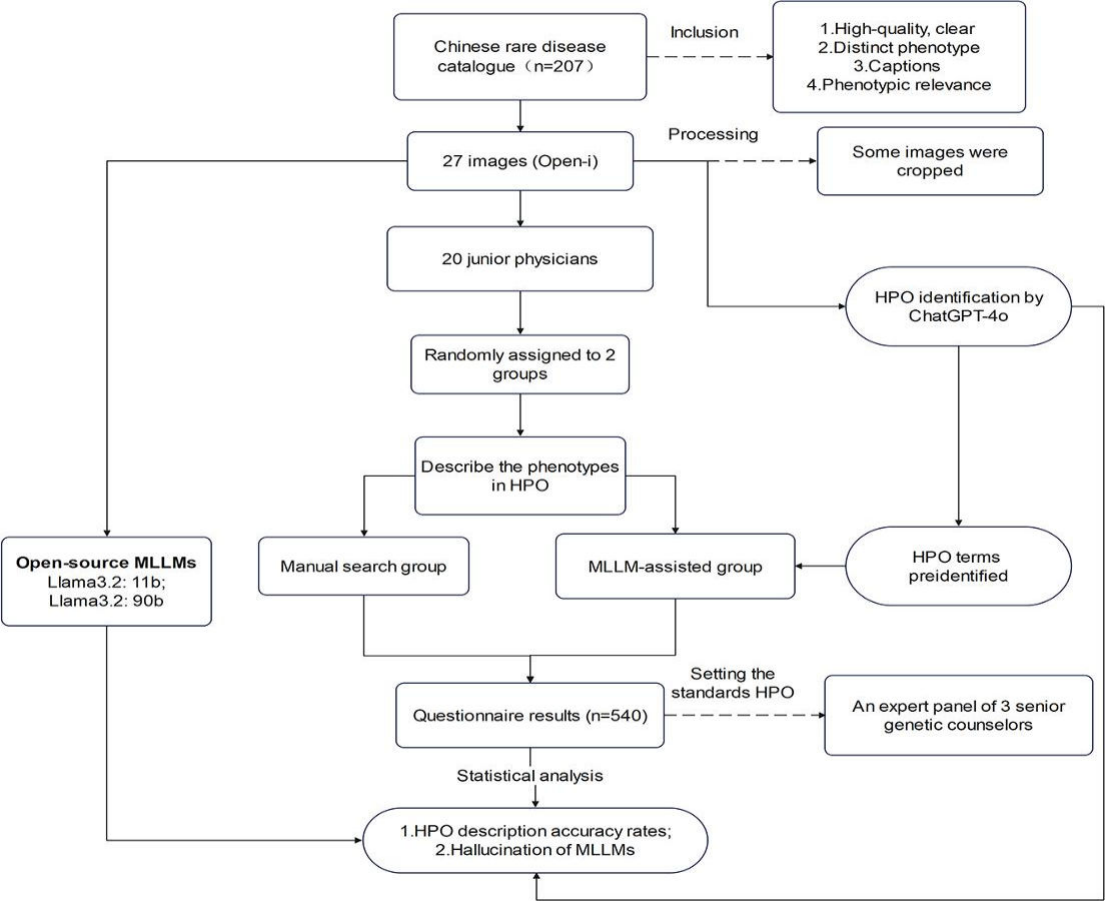
Overall flowchart of the study design. This figure illustrates the study’s methodology for evaluating the impact of MLLMs on the accuracy of HPO identification by junior physicians. Starting with a selection of 27 images from the Open-i database based on the Chinese Rare Disease Catalogue, the images were processed for quality and relevance. In total, 20 junior physicians were randomly divided into 2 groups: one using the Chinese Human Phenotype Ontology website for manual search and the other assisted by preidentified HPO terms from ChatGPT-4o. Additionally, ChatGPT-4o and 2 open-source MLLMs were tested for standalone HPO identification. An expert panel of genetic counselors established standard HPO terms for accuracy assessment. The study concluded with a statistical analysis of HPO description accuracy and MLLM hallucination rates based on questionnaire results from both groups. The Open-i service of the National Library of Medicine facilitates the search and retrieval of abstracts and images. HPO: Human Phenotype Ontology; MLLM: multimodal large language model.

To minimize the influence of varying clinical experience, the study recruited 20 junior physicians, including graduate students in clinical medicine and attending physicians, from diverse hospital backgrounds and specialties. Participants were evenly distributed across 10 fields: gynecology (n=2), obstetrics (n=2), gradiology (n=2), orthopedics (n=2), obstetrics and gynecology (n=2), surgery (n=2), reproductive medicine (n=2), pediatrics (n=2), internal medicine (n=2), and oncology (n=2). The study design is illustrated in Figures1  2.

Participants, who were colleagues, classmates, or acquaintances of the researchers, were tasked with providing detailed phenotype descriptions for 27 patient images sourced from public web-based platforms. They were compensated US $14 to ensure engagement and diligent completion of the task.

**Figure 2.**
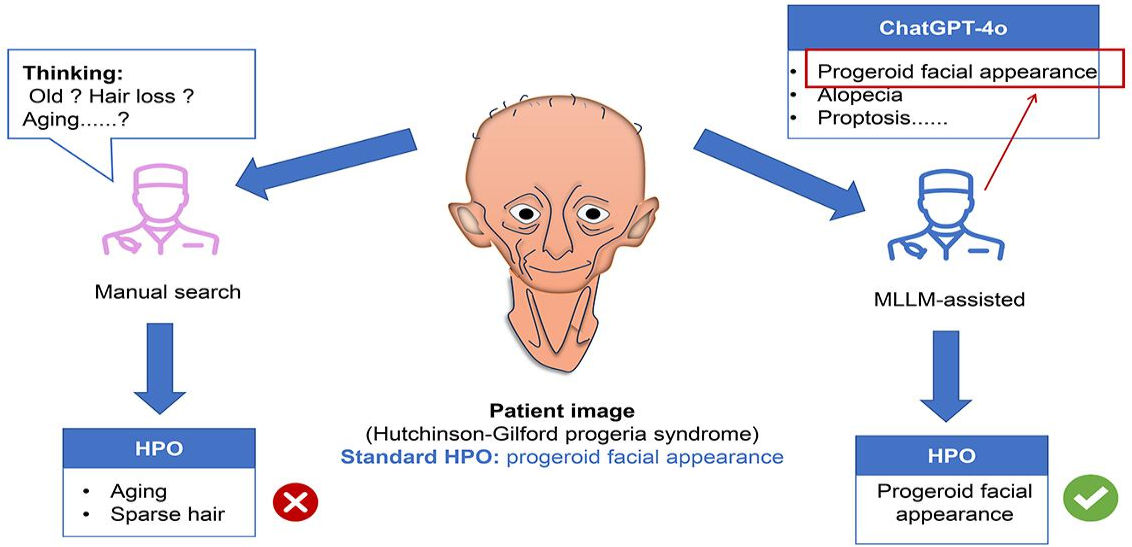
Workflow for patient image recognition using HPO by junior physicians in both cohorts. This figure depicts a sample image of a patient with Hutchinson-Gilford progeria syndrome. Both groups used only the image and their expertise to characterize the patient’s phenotype with HPO terms. The MLLM-assisted cohort further used pregenerated ChatGPT-4o prompts, potentially enhancing identification accuracy. Test images encompassed additional anatomical regions, including hands, legs, and skin. HPO: Human Phenotype Ontology; MLLM: multimodal large language model.

#### Group Stratification and Task Assignment

The 20 physicians were stratified by specialty and randomly assigned to 2 groups. Both groups evaluated the same 27 images through a web-based platform but used different approaches:

1. Manual search group: participants received a questionnaire and were instructed to use the Chinese Human Phenotype Ontology (CHPO) website [36] search for the most appropriate HPO to describe the phenotypes in the images. They were informed that multiple terms might apply to a single image. The CHPO was chosen for its ability to provide standardized HPO terms in Chinese, ensuring consistency across participants.
2. MLLM-assisted group: participants received a questionnaire where each image was accompanied by HPO terms preidentified by ChatGPT-4o. They were instructed to independently verify and search for appropriate HPO terms using the CHPO.

#### Measures to Prevent Bias

To prevent psychological bias, participants were not informed that 1 questionnaire included HPO terms identified by ChatGPT-4o. The original results of the 540 completed questionnaires can be found in Sheet 3 in Multimedia Appendix 1. Additionally, following the completion of the phenotype recognition questionnaire, we distributed a supplementary survey to the 20 clinicians to assess their background knowledge in rare diseases. The survey comprised the following items: years of clinical practice, number of rare diseases encountered annually, attendance at training sessions on rare and genetic diseases, prior awareness of HPO, HPO searches before the survey, and the necessity of describing patient phenotypes in clinical practice. More crucially, the survey also explored the factor they found most challenging in HPO image description after completing the questionnaire.

### Setting the Standards for HPO

Accurately determining HPO terms from 2D images is challenging due to factors such as camera angle, lighting, and pixelation, which can obscure phenotypic features. Additionally, the hierarchical nature of HPO terms introduces variability, as descriptions often depend on the describer’s knowledge and subjective interpretation.

To address these challenges, an expert panel of 3 senior genetic counselors from a prenatal diagnosis center—the chief physician, the deputy chief physician, and the deputy chief technologist of the center’s laboratory—was convened. All 3 are experienced geneticists with extensive HPO expertise. The panel systematically reviewed each patient image, paired with phenotype descriptions from the original literature, to interpret the patients’ phenotypes accurately. Through collaborative discussions and consultation with the CHPO, they established a set of correct HPO terms for each image. Despite the inherent subjectivity of HPO descriptions, disagreements were rare and typically resolved by prioritizing consensus. In cases of differing opinions, the majority view (2 of 3) prevailed, though contentious terms were retained for comprehensiveness. If no consensus emerged, the chief physician’s decision would take precedence—though this was not required in our study. A researcher then used the panel’s input to draft preliminary diagnostic criteria.

Following this, the junior physicians submitted 540 HPO terms across 27 images, which the panel evaluated after completing their questionnaires. Each image was presented in a slide format, displaying the preliminary diagnostic criteria alongside the 20 corresponding questionnaire responses. The panel reviewed these slides, synthesizing all data and resolving discrepancies through further discussion to finalize the standard HPO terms (Sheets 4 and 5 in Multimedia Appendix 1). This rigorous process ensured the accuracy and reliability of the HPO term standards, establishing the final “gold standard” for the study. The approach to handling disagreements remained consistent throughout, aligning with the method used for the preliminary criteria.

### Study Outcomes

The primary outcome was the accuracy of HPO descriptions provided by the 2 groups of junior physicians for patient images. A description was considered correct if it included all standard HPO terms for a given image; missing any standard term rendered it incorrect.

A strict requirement for complete consistency with standard HPO terms was intentionally avoided. This approach acknowledges the complexity of patient images, which may depict a wide range of phenotypic features, some of which may be insignificant or open to interpretation. In clinical practice, physicians’ observations are often subjective, and capturing the most critical phenotypic features—those essential for diagnosis and treatment—is sufficient. Thus, descriptions that included the most important HPO terms were deemed accurate. This assessment method was designed to reduce rigidity and better reflect the practical challenges of describing rare disease phenotypes in clinical settings.

### Statistical Analysis

Before recruiting junior physicians, 2 researchers independently provided HPO descriptions for all 27 patient images—one using manual search methods and the other using MLLM-assisted approaches. Preliminary estimates indicated an accuracy rate of 44% for manual search and 74% for MLLM-assisted. Sample size calculations were performed using R software (version 4.3.2; R Foundation for Statistical Computing) with the *pwr* package. With α=.05 and a power of 0.8, the minimum required sample size per group was approximately 41 (n=40.7). While 2 junior physicians per group evaluating the 27 images would meet this requirement, 20 physicians were recruited to include a broader range of specialties and minimize potential biases.

The Pearson chi-square test was used to compare HPO description accuracy rates between the 2 groups. Statistical significance was set at *P*<.05. The McNemar test was used to compare interspecialty variability between the 2 groups, specifically assessing differences in discordant pairs (cases where one group was correct and the other incorrect). The exact conditional McNemar test, implemented via the *exact2x2* package, was applied to ensure precision. The odds ratio, defined as the ratio of group 1-correct/group 2-incorrect cases (b) to group 1-incorrect/group 2-correct cases (c), was calculated to evaluate the relative likelihood of success for MLLM-assisted searches compared to manual searches among discordant pairs. To address zero-cell counts, the Haldane-Anscombe correction (adding 0.5 to both b and c) was used for odds ratio estimation. Since the statistical comparisons between the 2 groups of interspecialty variability were exploratory, no *P* value correction was applied.

In addition to physician performance, the accuracy of HPO identification by MLLMs was analyzed. Hallucination, such as discrepancies between HPO terms and IDs or the generation of nonexistent terms, was observed. However, the accuracy and hallucination rates of standalone MLLMs were secondary observations and not formally tested.

## Results

### Primary Outcome

The performance of the 2 physician groups in identifying correct HPO terms for the 27 patient images is summarized in Table 1. On average, 6.74 (SD 4.28) physicians in the MLLM-assisted group correctly described each image compared to 2.04 (SD 2.59) in the manual group.

Of the 270 descriptions collected from each group, the MLLM-assisted group achieved a correctness rate of 67.4% (182/270), while the manual group achieved 20.4% (55/270). Statistical analysis showed that the MLLM-assisted group’s accuracy in describing HPO terms was significantly higher than that of the manual group (*χ*^2^=121.3; *df=1,* relative risk 3.31, 95% CI 2.58-4.25; *P*<.001). A complete dataset of raw HPO term descriptions and final judgment outcomes is provided in Sheets 3 and 4 in Multimedia Appendix 1.

To quantify the clinical impact of MLLM assistance, we calculated the absolute risk reduction and number needed to treat. The absolute risk reduction was 47%, indicating 47% increase in accuracy with MLLM support. The number needed to treat was approximately 3, meaning that for every 3 HPO terms described, MLLM assistance led to 1 additional correct description.

**Table 1.** Correct counts of Human Phenotype Ontology (HPO) descriptions for 27 rare disease patient images by 2 groups of physicians^a^.

No	Rare disease	MLLM^b^-assisted (n=10), n (%)	Manual search (n=10), n (%)
1	Albinism	10 (100)	0 (0)
2	Alport syndrome	10 (100)	6 (60)
3	Amyotrophic lateral sclerosis	5 (50)	0 (0)
4	Angelman syndrome	10 (100)	0 (0)
5	Jeune syndrome	3 (30)	1 (10)
6	Congenital scoliosis	10 (100)	9 (90)
7	Fabry disease	1 (10)	0 (0)
8	Gaucher disease	10 (100)	0 (0)
9	Generalized myasthenia gravis	10 (100)	4 (40)
10	Hereditary angioedema	10 (100)	5 (50)
11	Marfan syndrome	10 (100)	4 (40)
12	McCune-Albright syndrome	10 (100)	6 (60)
13	Noonan syndrome	10 (100)	3 (30)
14	Peutz-Jeghers syndrome	10 (100)	2 (20)
15	POEMS^c^ syndrome	2 (20)	0 (0)
16	Achondroplasia	0 (0)	0 (0)
17	Acromegaly	0 (0)	0 (0)
18	Adult-onset Still disease	10 (100)	1 (10)
19	Alagille syndrome	0 (0)	0 (0)
20	Bardet-Biedl syndrome	4 (40)	6 (60)
21	Blue rubber bleb nevus	10 (100)	1 (10)
22	Cutaneous T-cell lymphomas	10 (100)	0 (0)
23	Fibrodysplasia ossificans progressiva	7 (70)	0 (0)
24	Generalized pustular psoriasis	0 (0)	0 (0)
25	Hidradenitis suppurativa	0 (0)	0 (0)
26	Hutchinson-Gilford progeria syndrome	10 (100)	4 (40)
27	Lennox-Gastaut syndrome	10 (100)	3 (30)

a The data in the table represent the number of physicians who correctly used HPO descriptions for the corresponding images.

b MLLM: multimodal large language model.

c POEMS: polyneuropathy, organomegaly, endocrinopathy, M-protein, skin changes.

### Departmental Variability in HPO Description Accuracy

**Figure 3.**
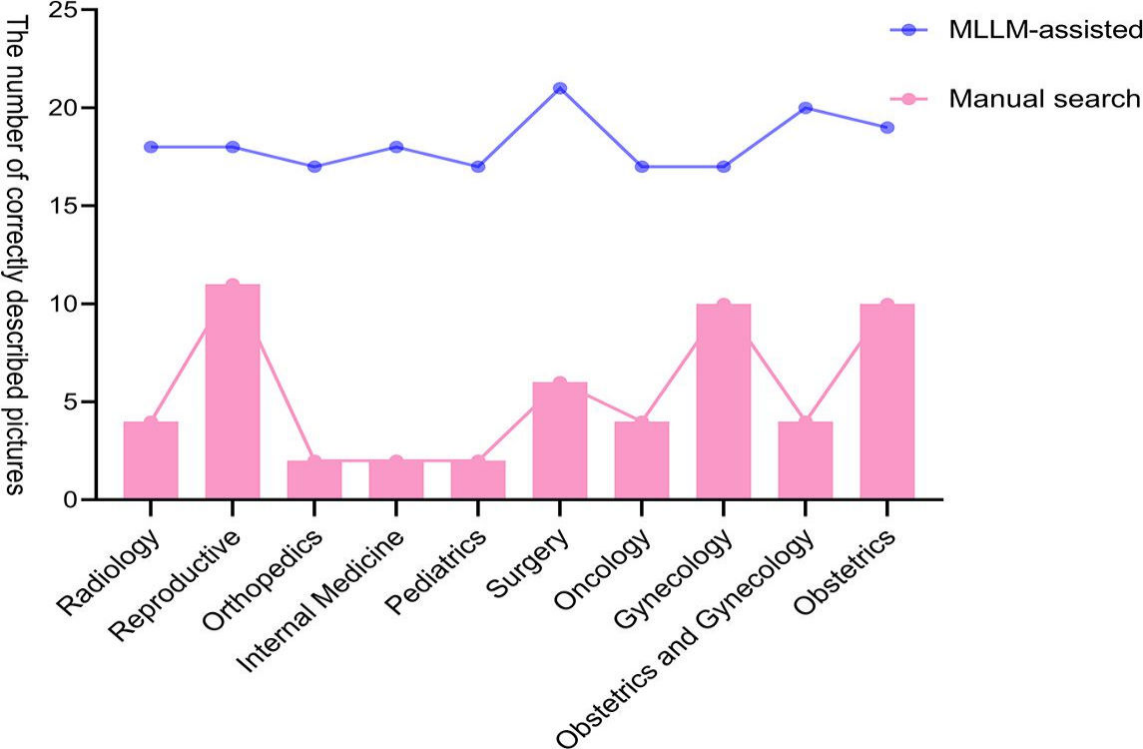
Accuracy of Human Phenotype Ontology (HPO) descriptions for patient images by junior physicians from different departments. Each department is represented by a single physician. In the manual search group, significant variability in the accuracy of HPO descriptions for patient images was observed among physicians from different departments. In contrast, in the MLLM-assisted group, all physicians achieved higher description accuracy compared to the manual search group, with more consistent performance levels across the group. MLLM: multimodal large language model.

The participating physicians represented 10 departments (Figure 3). In the manual group, HPO description accuracy varied significantly across departments. Physicians from reproductive medicine, gynecology, and obstetrics achieved the highest accuracy rates, while those from orthopedics, internal medicine, and pediatrics had the lowest. In contrast, the MLLM-assisted group showed more consistent accuracy across all departments, with correct counts consistently higher than those in the manual group.

Table 2 presents a comparison of interspecialty variability between the 2 groups, providing precise data to support Figure 3. Across all specialties, the MLLM-assisted group consistently demonstrated a higher description accuracy rate than the manual group, with statistically significant differences (*P*<.05). This finding reinforces our primary conclusion. However, due to the limited number of participating physicians per specialty, this analysis remains exploratory, and results should be interpreted with caution.

**Table 2.** Comparison of the interspecialty variability between the 2 groups.

Specialty	MLLM^a^-assisted (n=27), n (%)	Manual search (n=27), n (%)	OR^b^ (95% CI)^c^	*P* value
Reproductive medicine	18 (67)	11 (41)	5.7 (1.0-32.2)	.04
Gynecology	17 (63)	10 (37)	5.7 (1.0-32.2)	.04
Obstetrics	19 (70)	10 (37)	19.0 (1.1-326.5)	.04
Surgery	21 (78)	6 (22)	31.0 (1.9-518.1)	<.001
Radiology	18 (67)	4 (15)	29.0 (1.7-486.2)	<.001
Oncology	17 (63)	4 (15)	9.7 (1.8-51.9)	.001
Obstetrics and gynecology	20 (74)	4 (15)	33.0 (2.0-550.1)	<.001
Orthopedics	17 (63)	2 (7)	31.0 (1.9-518.1)	<.001
Internal medicine	18 (67)	2 (7)	33.0 (2.0-550.1)	<.001
Pediatrics	17 (63)	2 (7)	11.0 (2.1-58.5)	<.001

a MLLM: multimodal large language model

b OR: odds ratio.

c Adjusted ORs and their 95% CI were reported to ensure stability in small-sample settings.

### MLLMs Alone

The same methodology was used to evaluate the accuracy of HPO identification by standalone MLLMs without physician guidance. Among the 27 patient images, ChatGPT-4o achieved an accuracy rate of 48% (13/27), while Llama3.2:11b and Llama3.2:90b achieved rates of 15% (4/27) and 18% (5/27), respectively (Table 3).

Given the issue of hallucination in current LLMs, we reviewed the authenticity of the HPO terms generated (Sheet 6 in Multimedia Appendix 1). ChatGPT-4o produced 117 HPO terms, each with an HPO ID. Verification revealed that 57.3% (67/117) had incorrect IDs, and 34.2% (40/117) were fabricated, as they could not be retrieved on the HPO website.

Similarly, Llama3.2:11b generated 63 HPO terms, with only 1 term having a correct ID. Thus, 98% (62/63) had mismatched IDs, and 41% (26/63) were fabricated. Llama3.2:90b, despite its larger parameter size, produced 56 HPO terms, of which 82% (46/56) had incorrect IDs, and 32% (18/56) were fabricated.

**Table 3.** Accuracy of Human Phenotype Ontology (HPO) identification and frequency of hallucinations in patient images by multimodal large language models.

Category	ChatGPT-4o, n/N (%)	Llama3.2:11b, n/N (%)	Llama3.2:90b, n/N (%)
Identification			
Correct	13/27 (48)	4/27 (15)	5/27 (18)
Incorrect	14/27 (52)	23/27 (85)	22/27 (82)
Hallucinations^a^			
Incorrect IDs	67/117 (57.3)	62/63 (98)	46/56 (82)
Fabricated terms	40/117 (34.2)	26/63 (41)	18/56 (32)

a Hallucinations refer to model-generated HPO terms that either mismatch their IDs or are nonexistent.

### Background Information on Rare Diseases for Junior Physicians

Following the patient description questionnaire, we recontacted the 20 junior physicians and conducted a survey on their rare disease background. The survey assessed years of clinical practice, annual encounters with patients with rare disease, and training in rare and genetic diseases, among other factors (Sheet 7 in Multimedia Appendix 1). A baseline comparison of the junior physicians revealed a relatively balanced distribution between the 2 groups. The manual search group reported an average of 5.0 (SD 1.94) years of clinical practice compared to 5.3 (SD 2.11) years in the MLLM-assisted group. Physicians in the MLLM-assisted group encountered more patients with rare disease annually than those in the manual search group (mean 8.4, SD 5.72 vs mean 5.4, SD 3.06). The distribution of training and exposure to rare diseases, genetic diseases, and the HPO was also relatively balanced between groups. Due to the small sample size, no statistical tests were conducted.

To investigate why certain specialties outperformed others in the manual group, we ranked the physicians by accuracy rate and compared the top half (4 physicians, as the fifth physician’s accuracy rate of 14.8% matched the next 3) with the bottom half (6 physicians). The analysis suggested that attendance at rare disease training (n=2, 50% vs n=1, 17%) and genetic disease training (n=4, 100% vs n=4, 67%) may contribute to higher accuracy, as these factors were more common among the top performers.

Finally, we surveyed the 20 physicians on the challenges they faced when describing patient phenotypes. Only 1 (5%) physician cited unfamiliarity with anatomy as the primary difficulty, 6 (30%) reported difficulty finding suitable HPO terms, and the majority, 13 (65%), identified both issues as barriers to accurate HPO retrieval.

## Discussion

### Principal Findings

This study evaluated the accuracy of MLLMs in identifying terms from the HPO. The findings revealed that the MLLM-assisted group achieved significantly higher accuracy in describing HPO terms for patient images associated with rare diseases compared with the manual search group (182/270, 67.4% vs 55/270, 20.4%). Notably, there was substantial variability in the accuracy of descriptions among physicians from different departments in the manual group. In contrast, the MLLM-assisted group demonstrated consistently high performance regardless of departmental differences. When the performance of the MLLMs was evaluated independently, ChatGPT-4o achieved a description accuracy rate of 48% (13/27), outperforming the open-source models Llama3.2:11b and Llama3.2:90b, which had accuracy rates of 15% (4/27) and 18% (5/27), respectively. These results indicate that the highest level of accuracy in identifying HPO terms can be achieved through collaboration between junior physicians and MLLMs, combining human expertise with the computational capabilities of these models. Despite their promise, a notable limitation of current MLLMs is their high rate of hallucination in generated HPO terms. Many terms produced by the models either did not match official HPO IDs or were entirely fabricated.

### Limitations

This study has several limitations. First, the small sample size—20 physicians and 27 patient images—may limit the generalizability of the findings to broader clinical contexts. Second, the evaluation did not include several other MLLMs [37-39], which could have provided a more comprehensive performance comparison. Additionally, relying solely on publicly available patient images may not fully replicate real-world clinical phenotype description processes. There is also a possibility that some images were part of the models’ training datasets, potentially introducing bias. However, the study’s primary aim was not to diagnose conditions but to assess MLLMs’ ability to identify phenotypes using HPO terms. Thus, the evaluation of their performance on unseen images remains reasonably valid. Finally, our study did not include senior physicians, especially those adept in HPO searches and possessing deep anatomical expertise, likely achieve greater baseline accuracy in manual identification. Consequently, the difference in performance between manual and MLLM-assisted approaches may be less pronounced for these experts. Nonetheless, as clinicians with extensive rare disease experience are rare, our study targeted junior physicians to represent the wider clinical landscape and highlight MLLM’s potential to boost diagnostic precision.

### Comparison With Prior Work

Previous studies have shown that LLMs like ChatGPT excel in recognizing phenotype concepts in natural language and [10], with fine-tuning, outperform traditional tools in identifying HPO IDs [40]. However, research on MLLMs for HPO recognition remains limited, and their broader medical potential is underexplored [18,33]. While MLLMs have demonstrated success in tasks like pathological image classification [41] and chest radiograph diagnosis [18,42-44], their application to rare diseases is hindered by the lack of high-quality, disease-specific datasets [45,46]. Unlike earlier methods requiring extensive annotated medical images, ChatGPT-4o benefits from pretraining on diverse public datasets, making it particularly suitable for rare disease tasks. The survey on the rare disease knowledge of junior physicians indicates that attendance at rare disease and genetic disease training may contribute to improved performance among some physicians. This is plausible, as even in specialties with frequent exposure to patients with rare disease, physicians typically rely on nonstandardized natural language for descriptions and are confined to rare diseases within their own field. Training in rare and genetic diseases appears to enhance junior physicians’ ability to accurately describe patient phenotypes. However, even the highest-performing physicians in the manual search group achieved an accuracy rate of only 40.7%, which was surpassed by all physicians using MLLM assistance. The extensive anatomical knowledge embedded in MLLMs, combined with the provision of standardized HPO terms, provides valuable support for junior physicians. This assistance also mitigates the primary challenges most physicians reported in describing phenotypes.

Our study reveals that collaboration between junior physicians and ChatGPT-4o significantly enhances HPO description accuracy for rare diseases, outperforming both traditional methods and standalone MLLM use. This aligns with prior research showing the benefits of combining clinician expertise with visual-language models [47]. Phenotype-driven diagnostic strategies for rare diseases are widely acknowledged as effective [8,9]; yet, clinicians often face challenges in accurately describing phenotypes using standardized HPO terms during initial consultations. The complexity of human anatomical variations [48] and the extensive catalog of HPO terms pose significant barriers to precise documentation and communication. Our findings demonstrate that MLLM-assisted workflows streamline this process, improving accuracy and completeness over unaided methods. Additionally, MLLMs like ChatGPT-4o excel in processing contextual information, offering a clear advantage over traditional artificial intelligence tools [17,22]. For instance, in this study, when ChatGPT-4o was provided with the key contextual detail that a patient with albinism is of a person of color, it correctly identified “hypopigmentation of the skin” as a significant HPO. Without this context, the model might have incorrectly assumed the patient to be White, potentially overlooking this crucial skin phenotype.

Current MLLMs face several challenges in phenotype recognition tasks. First, they often generate irrelevant HPO terms, which can mislead physicians. For example, when analyzing an image of a patient with Bardet-Biedl syndrome, the model failed to identify the standard HPO term “polydactyly.” This led to a higher error rate in the MLLM-assisted group (6/10 physicians) compared to the manual group (4/10). This anchoring effect, caused by the model’s output, is unavoidable and can mislead some participants, diminishing their ability in certain specific diagnoses. While real-world clinical settings allow patients to clarify ambiguous findings, this example underscores the risk of models inadvertently misleading clinicians. Additionally, the accuracy of HPO identification remains suboptimal. ChatGPT-4o, the top-performing model, achieved only 48% (13/27) accuracy, with open-source models performing even worse. This highlights the early developmental stage of MLLMs in HPO identification and the significant performance gaps among models from different vendors [29,49,50]. Moreover, as observed in prior research, the phenomenon of hallucination persists in MLLMs [17,27]. These models frequently fabricate HPO terms or generate IDs that do not correspond to actual entries, further limiting their applicability in clinical workflows. Although open-source models are often preferred for patient privacy protection, their performance and hallucination rates in this study were concerning, likely due to limited pretraining on medical images and HPO-related materials [33]. Even a large open-source model with 90 billion parameters lagged significantly behind ChatGPT-4o in phenotype identification. However, open-source models hold promise for future applications. With ongoing technological advancements and task-specific fine-tuning, their performance and reliability can be improved.

In this study, we contend that the risks posed by hallucinations in MLLMs for HPO identification are less severe than those associated with LLMs for direct diagnosis. We classified hallucinations into 2 categories: mismatched HPO IDs and fabricated, nonexistent HPO terms. When MLLMs are used solely to aid in describing patient features within medical records, these hallucinations are unlikely to exert a substantial adverse effect. In the context of integrating generated HPO terms with phenotype-driven diagnostic tools, an accurate HPO name ensures that mismatched IDs do not compromise the diagnostic process. Fabricated terms, being unrecognizable by such tools, may simply prompt researchers to identify correct terms or exclude the erroneous ones. Given that phenotype-driven tools provide reference-based diagnostic outcomes, with confirmatory diagnoses typically relying on genetic testing, this approach demonstrates greater clinical acceptability than direct LLM-based diagnosis.

While MLLMs can augment junior physicians’ ability to describe patient images, their outputs require careful application in clinical settings. Clinicians must leverage their expertise to distinguish reliable content from inaccuracies. Furthermore, when MLLM outputs contribute to medical record documentation, oversight by seasoned physicians is essential. Finally, for academic discourse or use in phenotype-driven diagnostic tools, generated HPO terms should be validated against the HPO database to ensure accuracy.

### Conclusions

The integration of MLLMs into clinical workflows demonstrates potential in enhancing junior physicians’ ability to describe disease phenotypes using standardized HPO terms. This collaborative approach surpasses standalone MLLMs, underscoring the added value of physician involvement. While open-source MLLMs show promise in phenotype identification, even advanced models like ChatGPT-4o face challenges such as identification errors and hallucinated outputs. Future efforts should focus on fine-tuning open-source MLLMs with expanded datasets of diverse phenotype images and HPO-related corpora. This strategy could improve model accuracy, reliability, and patient privacy, ultimately facilitating more precise use of HPO in clinical practice and medical research.

## Supplementary material

10.2196/73233 Multimedia Appendix 1 The supplementary material contains 7 sheets, providing detailed information about the study. The specific content of each sheet is as follows: Sheet 1. Information on Open-Access images used for phenotype descriptions; Sheet 2. Chat logs output by multimodal large language models; Sheet 3. HPO terms filled in the questionnaires by the 2 Groups of Junior Physicians; Sheet 4. Accuracy judgments of HPO terms in the questionnaires; Sheet 5. Standard HPO answers established by the expert panel; Sheet 6. Hallucination of multimodal large language models; Sheet 7. Background information on rare diseases for junior physicians.

## Abbreviations

CHPO: Chinese Human Phenotype Ontology
HPO: Human Phenotype Ontology
LLM: large language model
MLLM: multimodal large language model
